# Endovascular repair of acute vs. subacute uncomplicated type B aortic dissection: a systematic review and meta-analysis

**DOI:** 10.3389/fcvm.2023.1189750

**Published:** 2023-07-12

**Authors:** WenXin Zhao, Yang Yang, ZhiYuan Wu, ZuoGuan Chen, YongPeng Diao, Yong Lan, YongJun Li

**Affiliations:** ^1^ Department of Vascular Surgery, Beijing Hospital, National Center of Gerontology, Institute of Geriatric Medicine, Chinese Academy of Medical Sciences, Beijing, China; ^2^ Graduate School of Peking Union Medical College, Chinese Academy of Medical Sciences, Beijing, China

**Keywords:** TEVAR, uncomplicated type B aortic dissection, acute, subacute, endovascular repair

## Abstract

**Objective:**

This study aimed to conduct a meta-analysis evaluating the optimal timing for endovascular repair of acute versus subacute uncomplicated Type B Aortic Dissection.

**Method:**

PubMed, EMBASE, web of science and Cochrane Library was interrogated to identify Electronic bibliographic studies updated to January 2023 to collect studies compared the clinical outcomes of endovascular repair for Acute Versus Subacute Uncomplicated Type B Aortic Dissection. Data were aggregated as pooled odds ratios (OR) using the fixed or random effects models according to the significance of heterogeneity, Pooled odds ratios (OR) were calculated by RevMan 5.3 and applied with fixed or random-effect models.

**Result:**

A comprehensive literature search found 322 citations published and finally among them 6 studies containing 3,769 patients (acute group 2,642, subacute group 1,127) were included in review. There is an increased risk of 30-day complications (OR = 1.51,95%CI,1.26–1.81) 30-day mortality (OR = 2.39,95%CI, 1.55–3.67) and 1-year mortality (OR = 1.71,95%CI,1.27–2.30) for an acute uTBAD group compared to subacute ones. Similarly, reintervention was more likely in the acute group than in the subacute group (OR = 1.42,95%CI,1.05–1.91). However, no significant differences were found in long-term mortality.

**Conclusion:**

This meta-analysis confirmed that there was no significant difference in the long-term prognosis between the acute and subacute phases in the timing of surgery. However, considering the high incidence of complications, high re-intervention rate and one-year mortality probably caused by high intima fragility in the acute phase, endovascular repair at subacute phase appears to favorably compare with acute strategy. But future studies with adequate patient numbers and longer-term follow-up are necessary to further verify the study conclusion.

**Systematic Review Registration:**

https://www.crd.york.ac.uk/prospero/display_record.php?ID=CRD42021247609, identifier PROSPERO CRD42021247609.

## 1. Introduction

Type B aortic dissection (TBAD) is a life-threatening condition with high morbidity of approximately 3 in 100,000 people (1), and of whom more than 60% present have no signs of rupture or malperfusion [termed uncomplicated TBAD (uTBAD)] (2, 3). Thoracic endovascular aortic repair (TEVAR) has been now recommended as first lifesaving option for patients with TBAD in the setting of complications (4–6) according to recent guidelines (7–10). As it is traditionally recommended that uTBAD be managed through optimal medication (OMT) (9, 11), TEVAR is rarely used to treat uTBAD in actual clinical work, which leads to limited research on it in uTBAD. But OMT remains bad compliance (12) and also has a high incidence of late aorta-related complications. Considering that drug therapy accompanied by poor long-term prognosis, high aneurysm variability and low late survival rate (13, 14), more and more doctors have begun to accept the application of endovascular intervention in the treatment of acute uTBAD, so as to achieve survival benefits by preventing late complications (8, 15).

As increasing use of TEVAR in uTBAD, the complications, mortality and reintervention seems to be related to the timing of treatment (16, 17). The related Research confirmed that aortic remodeling after TEVAR is a continuous process, and chronic dissections have a more rigid intimal flap resulting in slower remodeling than in acute dissection (18–20). However, the complication rate is higher in the acute phase caused by the higher intima fragility in this phase (21). We sought to determine the optimal intravascular “treatment window” to achieve the goal of maximizing the survival benefit of early preventive TEVAR by ensuring better utilization of aortic remodeling and minimizing the incidence of complications (16). At present, relevant studies have not reached a consistent conclusion. A retrospective multicenter study analyzed the impact of timing on survival and postoperative complications following TEVAR for uTBAD suggest that the patients receiving treatment in subacute period is associated with improved 30-day and 1-year survival (21), which is the reverse of Gupta and colleagues' conclusion (22). Therefore, the aim of this systematic review and meta-analysis was to obtain the optimal timing.

## 2. Methods

### 2.1. Systematic review and search strategy

This study was performed in accordance with the recommendations in the Preferred Reporting of Systematic Reviews and Meta-Analysis (PRISMA) statement (23). A search of PubMed, EMBASE, web of science and Cochrane Library was interrogated to identify Electronic bibliographic studies updated to January 2023. Our search string was as previously: (“stent” OR “endovascular”) AND (“DeBakey III” OR “type B”) AND “uncomplicated” AND “aortic dissection” AND (“timing” OR “phase” OR “period”) (24). Only English language articles were considered due to limited funding for translation and We also searched the reference lists of included studies and reviews for relevant reports.

### 2.2. PICO question

The population, intervention, comparison, outcome (PICO) question was: Specifically, “P” (**Participants/population**) refers to the type of patients studied**:**patients with uncomplicated stanford type B aortic dissection; “I” (**Interventions/exposures)** refers to Thoracic endovascular aortic repair (TEVAR) is the intervention; “C”(**Comparators/control)** refers to Timing of thoracic endovascular aortic repair for patients with uncomplicated acute type B aortic dissection and “O” (**Main outcomes)** refers to The main outcome is mortality or long-term survival.

### 2.3. The inclusion and exclusion criteria

According to the preferred reporting items of the systematic review and meta-analysis report published in 2009 (25), we set out the inclusion and exclusion criteria as following.

The inclusion criteria:

• Studies providing data for postoperative outcomes of TEVAR utilized to treat patients with acute uTBAD and subacute uTBAD
• Studies not only clearly define the uncomplicated and high-risk features (HRF) TBAD but also type A dissections or a combined hybrid endovascular or open thoracic aorta repair or complicated TBAD was definitely excluded from Study participants

The exclusion criteria:

• Studies with less than 100 patients were excluded.
• Literature based on study type, namely case reports, case series, one-arm studies, and literature reviews were excluded.
• Articles containing insufficient data <25% of predefined variables extractable were excluded.

In the cases of the same population of patients were identified or if study populations overlapped, only the most detailed or the latest reports were included to avoid duplication of data, unless the outcomes were mutually exclusive.

### 2.4. Data extraction

Titles and abstracts were reviewed independently for suitability based on the inclusion criteria by two authors (ZWX and YY). The full texts of suitable studies were independently assessed and related data extraction was performed independently by the same reviewers. When disagreement occurred, a third author (LY) was resorted to resolve the controversy. Information extracted from each study included the following: basic information about the included studies, such as first author, publication year, number of patients, baseline characteristics, recruitment period, short- and mid-term follow-up data and long-term follow-up results. For dichotomous data, the odds ratio (OR) and 95% confidence intervals (CI) were calculated using the base data reported in the frequency tables for each study.

### 2.5. Definitions

“Uncomplicated” was characterized as a dissection with no evidence of rupture or end-organ malperfusion (26). “High risk” was defined as patients with high-risk radiographic features, including initial false lumen diameter of 22 mm, a maximum aortic diameter of 40 mm, a patent or partially thrombosed FL, and an initial entry tear of 10 mm (27). Late reintervention was defined as any endovascular repair or open surgery in order to deal with dissection-related adverse events 30-day after the initial intervention. The complications occurred in hospital stay was classified into 30-day complications included aortic rupture, organ failure (renal failure and heart failure), heart complications (myocardial infarction and congestive heart failure), renal ischemia, respiratory complications, endoleak, neurological complications (spinal cord ischemia, paraplegia, and dialysis).Cooperating both the IRAD (28) and European Society of Cardiology findings (7), uncomplicated B aortic dissection has been categorized into: hyperacute, <24 h; acute, 1 to 14 days; subacute, 15 to 90 days; and chronic, >90 days.

### 2.6. Risk of bias

Quality assessment was conducted by the Newcastle-Ottawa scale (29). We evaluate these non-randomized study according to the representativeness of study samples, exposure ascertainment, blinding of outcome assessors, and loss to follow-up. The studies were then assigned as “low risk,” “high risk,” or “unclear” based on the risk of bias.

### 2.7. Statistical analysis

Appling with fixed or random-effect models, Pooled odds ratios (OR) estimates with corresponding 95% Cis which is the combined odds ratio value from each research were calculated by RevMan 5.3. Heterogeneity among studies was assessed by using the *χ*² and *I*^2^ tests.Funnel plot, Begg's and Egger's test were used to assess publication bias by Stata version 12.0. The t-test was used to compare the continuous variables between the two groups, and the *χ*2-test was used to compare the categorical variables.

## 3. Result

### 3.1. Study selection

**Figure 1:**
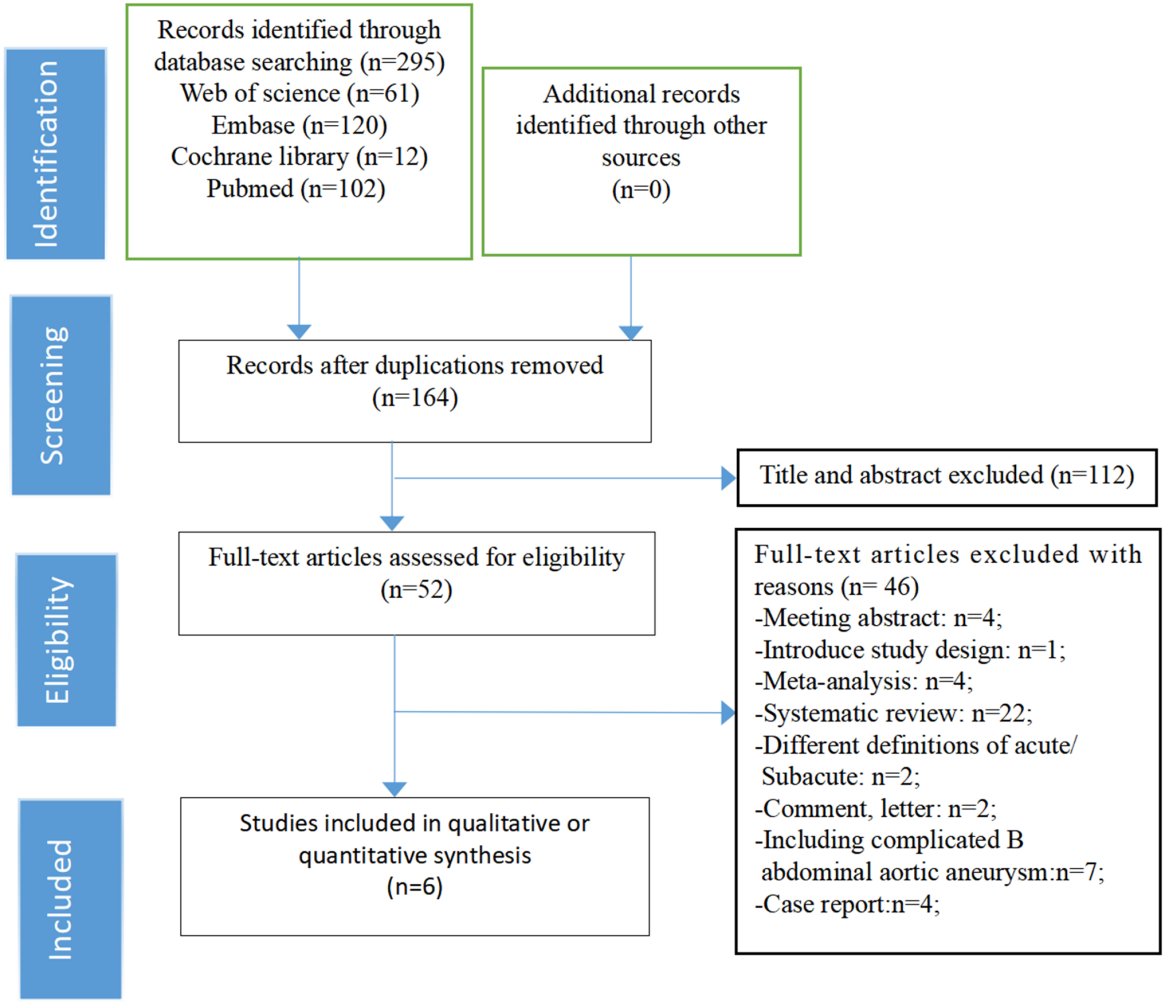
Flow diagram of study selection.

**Figure 2:**
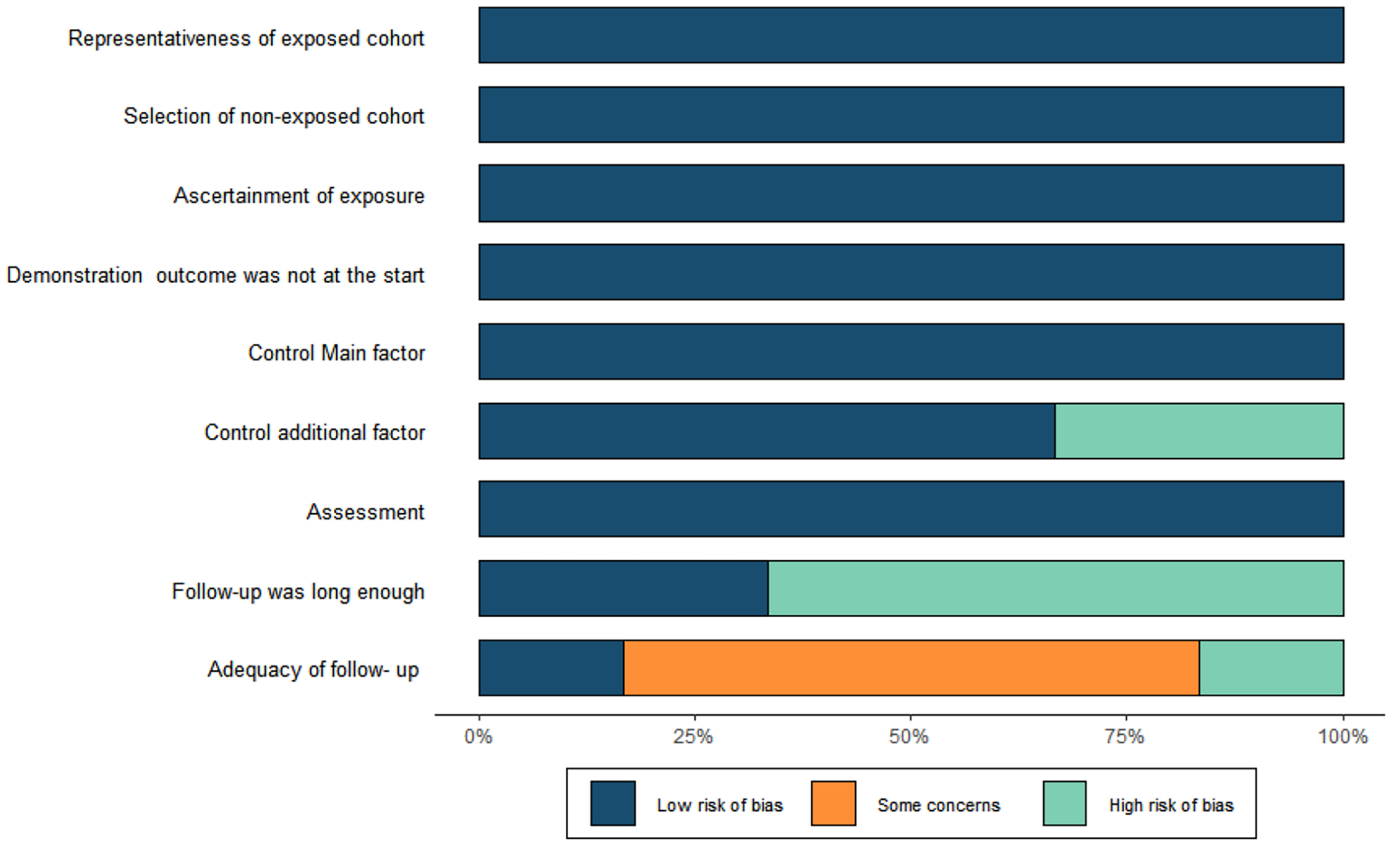
Risk of bias. Methodological Quality assessment of cohort studies based on the Newcastle-Ottawa Scale of each studies.

**Figure 3:**
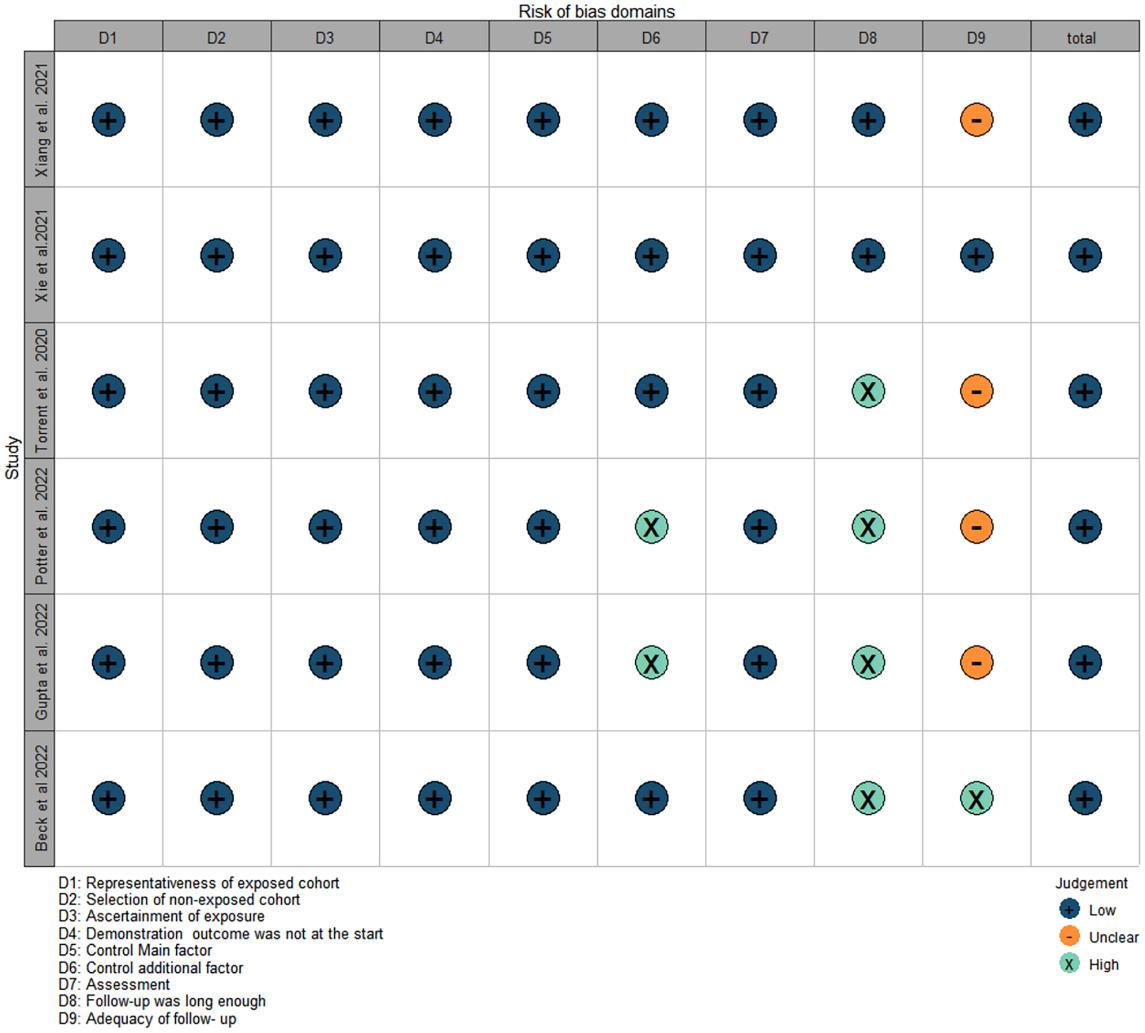
Risk of bias. Methodological Quality assessment of cohort studies based on the Newcastle-Ottawa Scale of each studies.

Through a comprehensive literature search, we initially obtained 295 relevant articles. After excluding duplicate literature and screening the titles and abstracts, 52 articles remained. When carefully review of the full text, we excluded an additional 46 articles based on the reasons listed in the figures. Only 6 studies ultimately met all the eligibility criteria were retained and were included in the meta-analysis (Figure 1). All of them were non-randomized, retrospective studies.The total number of patients included in the analysis was 3,769 and 2,642 patients were categorized as acute uTBAD and1127 as subacute uTBAD. The basic clinical characteristics of patients, including the number of patients, Recruitment period, 30 d complications and other basic clinical characteristics are summarized in the Table 1. Two authors (CZG, ZWX) independently assessed the methodological quality of the selected studies using the Newcastle-Ottawa Scale for retrospective studies (Figure 2). The thorough Newcastle-Ottawa Scale core >6 (6 of 6 studies), all indicate high quality of including studies (Figure 3).

**Table 1:** Characteristics of included studies.

Study/author year	Recruitment period (Year)	Timing of repair	Patient (*n*)	Male (*n*)	Mean age	30-day Complications (*n*)	30-day mortality (*n*)	Late Reintervention (*n*)	Late mortality (*n*)
Xiang et al. (30) 2021	2008–2018	acute	142	119	52.9	25	2	n.a	10
		subacute	96	76	52.8	9	0	n.a	9
Xie et al. (31) 2021	2010–2017	acute	130	107	55.7	19	5	6	5
		subacute	137	115	56.0	15	1	2	11
Torrent et al. (32) 2020	2010–2019	acute	446	278	60.7	103	26	39	39
		subacute	242	160	59.9	41	5	19	14
Potter et al. (33) 2022	2014–2020	acute	841	n.a	n.a	227	55	75	94
		subacute	259	n.a	n.a	50	7	17	12
Gupta et al. (34) 2022	2014–2020	acute	954	582	61.8	255	57	69	127
		subacute	316	208	60.8	60	8	12	24
Beck et al. (35) 2022	n.a	acute	129	84	60.4	24	4	7	10
		subacute	77	54	61.7	14	4	7	14

### 3.2. Risk of bias of included studies

We summarized the baseline characteristics of the selected patient population, and the results are shown in Table 2. There was no difference in the hypertension, coronary artery disease, cerebrovascular disease, renal insufficiency between patients receiving interventions during acute uTBAD and subacute uTBAD (*p* > 0.05). However, We found a difference in smoking (*p* = 0.0071) and COPD (*p* = 0.0419)between the two groups, which may lead to a bias in long-term prognostic outcomes.

**Table 2:** Patient characteristics.

	Acute (*n*/*n*)	Subacute (*n*/*n*)	*P*-value
Smoking	701/1,801 (38.92%)	291/868 (33.53%)	0.0071
Hypertension	1,055/1,801 (86.29%)	547/868 (88.13%)	0.1851
Coronary artery disease	229/1,801 (12.72%)	101/868 (11.64%)	0.4516
Cerebrovascular disease	47/847 (5.55%)	34/552 (6.16%)	0.6409
Renal insufficiency	164/1,801 (9.11%)	71/868 (8.18%)	0.4661
Chronic pulmonary disease	284/1,801 (15.77%)	111/868 (12.79%)	0.0419

### 3.3. Meta-analysis of 30-day complications and mortality

**Figure 4:**
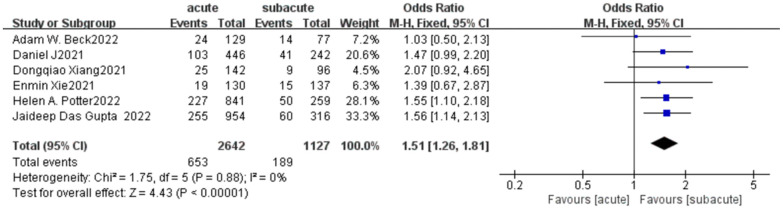
A 30-day complications forest plot of comparison of TEVAR for patients with acute vs. subacute uTBAD.

There were all 6 retrospective nRCTs included. A total of 3,769 patients were included in this part of study. Among them, 2,642 underwent EVAR in acute and the remaining in subacute. The incidence of a 30-day complications in patients with acute uTBAD was significantly higher than that in patients with subacute uTBAD (24.72% vs. 16.77%, OR = 1.51, *p* < 0.00001, Figure 4). *I*^2^ (0%) suggested there was little heterogeneity among included studies.

**Figure 5:**
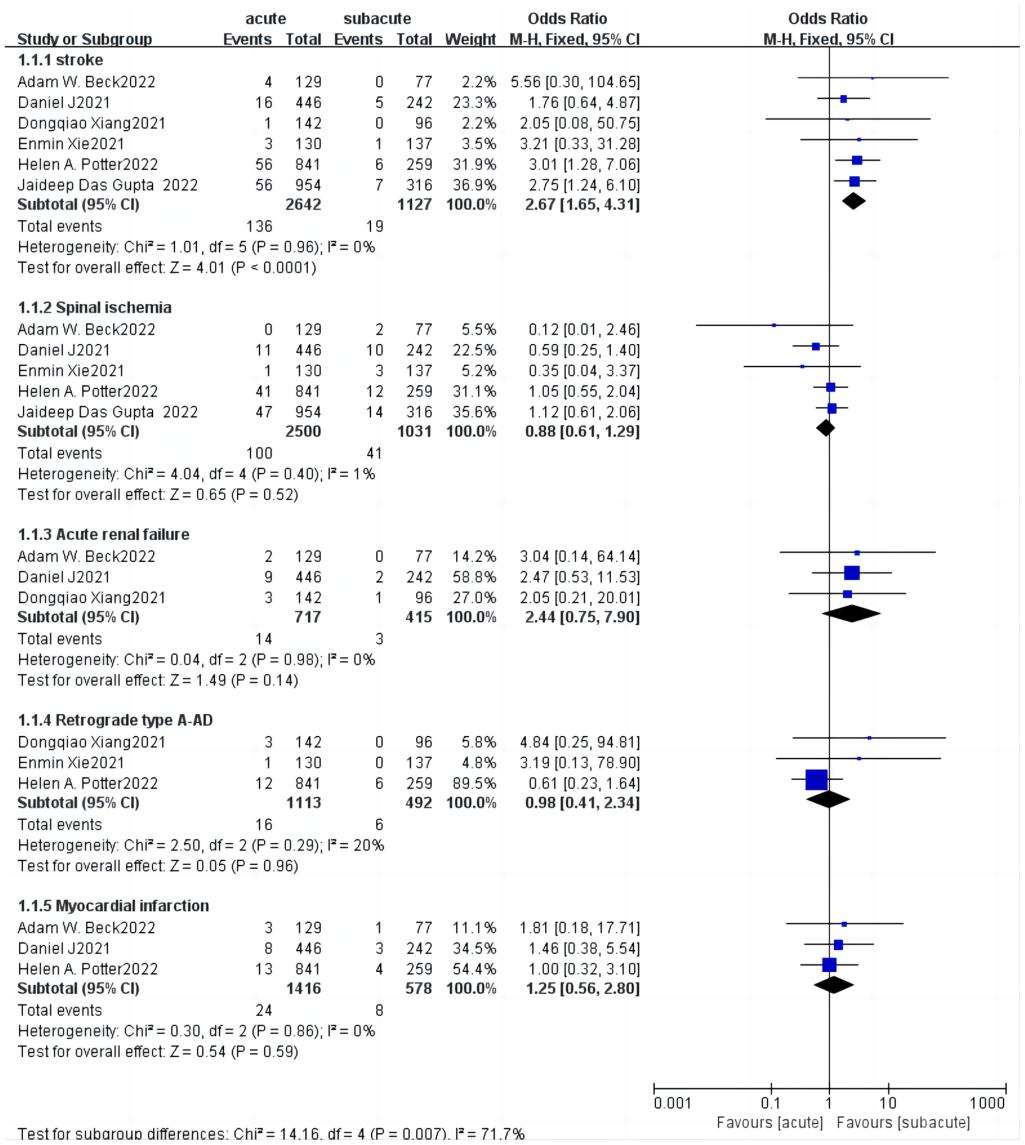
Subgroup analysis for 30-day complications forest plot of comparison of TEVAR for patients with acute vs. subacute uTBAD.

**Figure 6:**
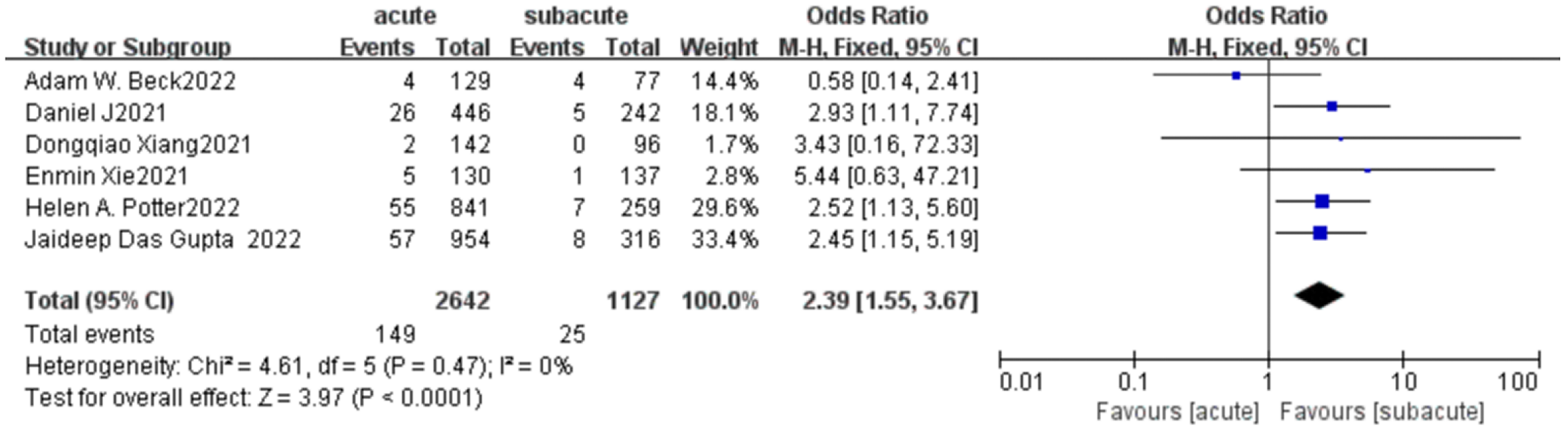
A 30-day mortality forest plot of comparison of TEVAR for patients with acute vs. subacute uTBAD.

In addition, we summarized and analyzed several common and widely valued complications in more detail shown in Table 3, and carried out meta-analysis of them. In all 30-day complications reported in these studies, except for spinal cord ischemia and retrograde type A dissection, the trend was toward higher rates of complications in the acute phase, with the incidence of cerebral ischemia significantly higher in patients with acute uTBAD than in the subacute phase (5.15 vs. 1.69%; OR = 2.67, *p* < 0.0001), but no significant difference was found in other complications (Figure 5). There was a significant increase in 30-day mortality in patients with acute uTBAD (3.95 vs. 0.66%; OR = 2.39, *p* < 0.0001; Figure 6). *I*^2 ^< 50% in all suggested low heterogeneity between studies.

**Table 3:** 30-day complications and mortality.

	Acute (*n*/*n*)	Subacute (*n*/*n*)	*P*-value
30-day complications	653/2,642 (24.72%)	189/1,127 (16.77%)	<0.0001
Aortic rupture	4/272 (1.47%)	0/233 (0.00%)	0.1280
Myocardial infarction	24/1,416 (1.69%)	8/578 (1.38%)	0.6984
Acute renal failure	14/717 (1.95%)	3/415 (0.72%)	0.1296
Type I endoleak	25/272 (9.19%)	15/233 (6.44%)	0.3215
Spinal cord ischemia	100/2,500 (4.00%)	41/1,031 (3.98%)	>0.9999
Stroke	136/2,642 (5.15%)	19/1,127 (1.69%)	<0.0001
Retrograde type A-AD	16/1,113 (1.44%)	6/492 (1.22%)	0.8199
30-day mortality	149/2,642 (3.95%)	25/1,127 (0.66%)	<0.0001

### 3.4. Meta-analysis of reintervention

**Figure 7:**
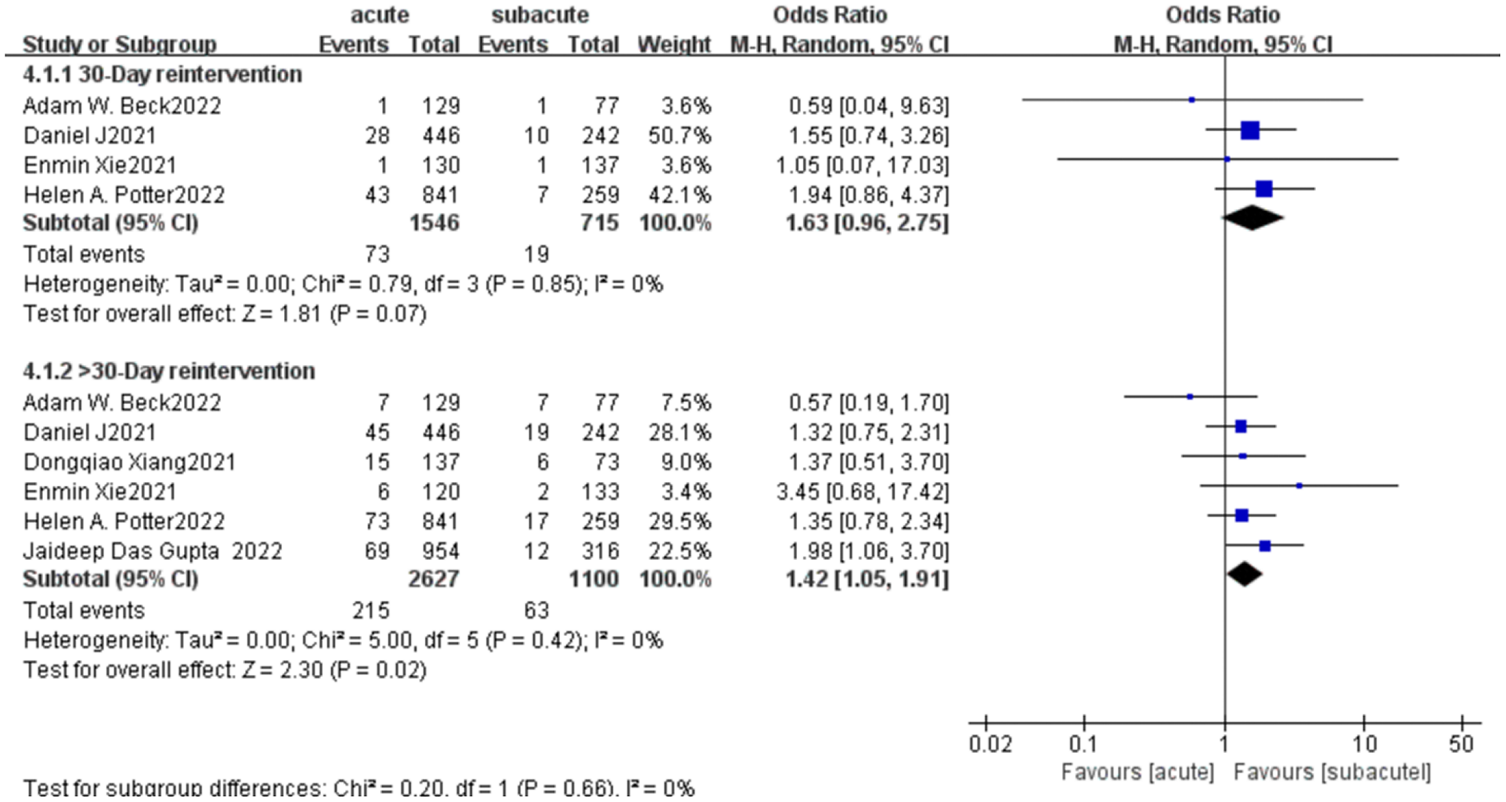
Reintervention forest plot of comparison of TEVAR for patients with acute vs. subacute uTBAD.

Of the 6 included papers, 4 included data on reintervention within 30 days, including 1,546 patients who received EVAR in the acute phase and 715 patients who received subacute treatment. The results of the meta-analysis suggested that there was no significant difference in the actual choice of surgery for early re-intervention (OR = 1.63, *p* = 0.07; Figure 7). In the late reintervention, the possibility of reintervention in the acute group was higher than that in the subacute group, suggesting that EVAR treated in the acute stage associated with a higher rate of late reintervention(OR = 1.42, *p* = 0.02; Figure 7). *I*^2^ (0%) suggested minimal heterogeneity between studies.

### 3.5. Meta-analysis of long-term mortality

**Figure 8:**
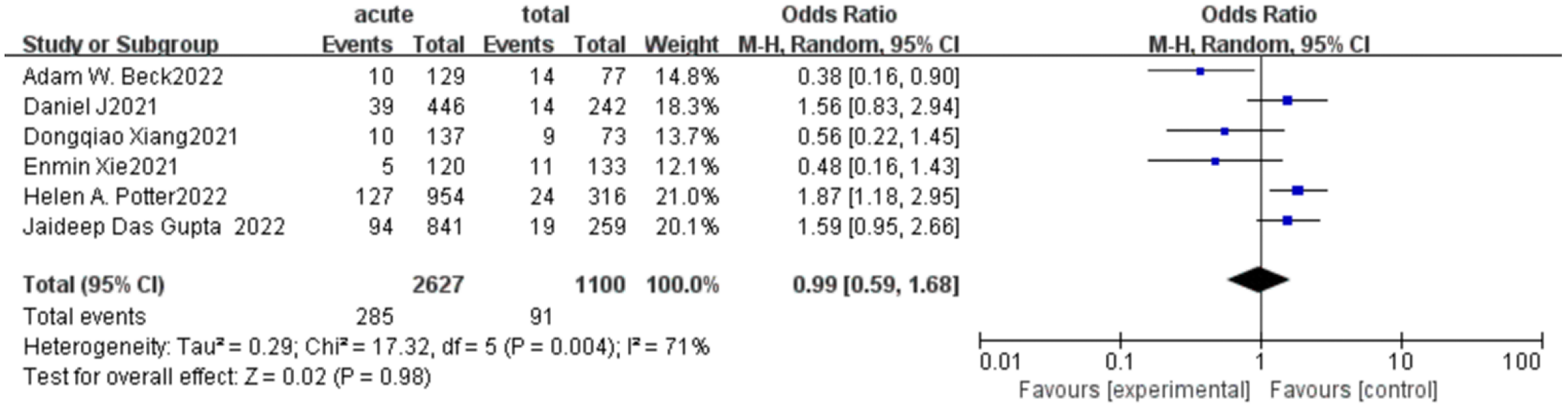
Long-term mortality forest plot of comparison of TEVAR for patients with acute vs. subacute uTBAD.

**Figure 9:**
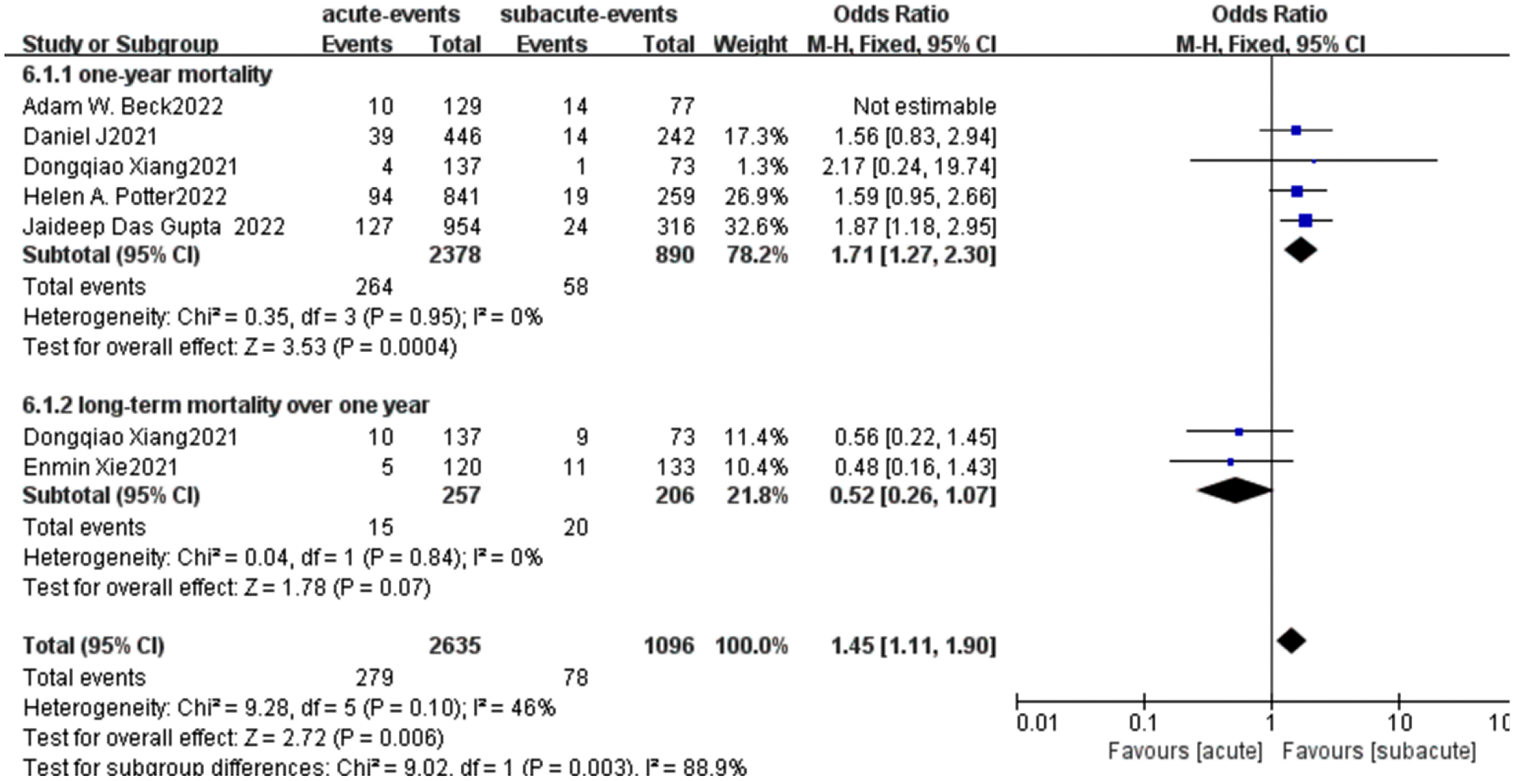
Subgroup analysis for long-term mortality forest plot of comparison of TEVAR for patients with acute vs. subacute uTBAD.

When we conducted a meta-analysis of long-term mortality rates for all 6 articles, we found that a high heterogeneity in long-term mortality (Chi^2^ = 17.32 P = 0.004, *I*^2^ = 71%; Figure 8). So we employed the random effect model and obtained the combined effect size OR = 0.99(*P* = 0.98) suggesting No significant difference in late mortality. To further analyze the sources of heterogeneity we performed a subgroup analysis, dividing the data into two groups based on the duration of follow-up: one-year mortality and mortality over one year. Interestingly, we found that the subgroup heterogeneity was significantly reduced (*I*^2 ^= 0) after the deletion of the study by Adam W. Beck which was highly sensitive, and the effect value of mortality within one year was OR = 1.71(*P* = 0.0004) (Figure 9), suggesting that the acute group was associated with a higher one-year mortality. In the long-term mortality over one year, we found that there was no statistical significance between the two groups, and the effect value OR = 0.52 suggesting the acute group had a relatively low mortality. It can be inferred that the survival benefit of the acute group increased with the extension of follow-up time.

### 3.6. Funnel plots and sensitivity analyses

All Funnel plots of meta-analyses were symmetric. All sensitivity analysis of meta-analyses indicated that the results were not dependent on inclusion of a single indicator (data Supplementary Figures).

## 4. Discussion

With the release of the Long-term results from studies such as INSTEAD-XL (36), prospective multicentre ADSORB trial (37), consensus on treatment of uncomplicated type B aortic dissection, seems to be challenged. Default medical management with focus on blood pressure and surveillance is no longer the only treatment, and more aggressive early intervention is widely accepted (38, 39). Jubouri et al., in a recent review, summarized a number of studies and found that long-term aortic remodeling is affected by the timing of intervention and different areas of dissection (40). Tadros et al. in their review mentioned that there was no difference in the regression of false lumen between the subacute and acute groups in patients with uncomplicated type B aortic dissection who receivingTEVAR. However, patients treated in the subacute phase had fewer complications such as RTAD and aortic rupture (15). Several studies have compared aortic remodeling, clinical outcomes, and procedure-related complications in uTBAD patients treated with TEVAR in the acute and subacute phases, and found that there was no statistically significant difference among timing groups in early and late outcomes (31–35). However, there is a significantly higher trend in perioperative complication rate, reintervention rate and mortality rate in the acute phase which should be a concern. The latest guidelines offer some advice, but do not specify when it is the optimal “therapeutic window” to do TEVAR. So far, studies on the timing of TEVAR are varied. Apart from a study conducted by our group in 2022, no other relevant meta-analyses have been published (24). Due to the limited number of included studies, the results of the previous meta-analysis were not significant and the evidence level was low. As a result, with the further development of related research, it is necessary to update and summarize the literature in time. herein, We try to perform a systematic review and meta-analysis aimed to identify the optimal timing of treatment by analyzing and evaluating the available evidence. Our study showed that the early outcome of TEVAR in the subacute uTBAD group was better than that in the acute group, while there was no significant difference in the late outcome, suggesting that the subacute stage seems to have emerged as the optimal time window for TEVAR in uTBAD.

We combined the results about 30-day complications and mortality of each study and obtained OR values by the fixed effects model, suggesting that the perioperative complications (*p* < 0.0001) and mortality (*p* < 0.0001) rates in the acute group were both more than twice as high as those in the subacute group, with a high statistical significance in both cases. Compared with the conclusion that had no statistical significance among two groups, the meta-analysis made use of statistical advantages and obtained a more clear conclusion through the increase of sample size. The significant increasing risk of complications in the acute phase should be owed to the fact that dissecting membranes are usually thinner and more fragile than usual (41). Acute surgical intervention aggravated the damage to the delicate intima of the inflamed aorta (21). Further analysis of common perioperative complications of general concern in aortic dissection showed that stroke was more than twice as high as those in the acute phase than in the subacute phase (*p* < 0.0001). Stents covering left subclavian artery (LSA) during TEVAR were associated with increased incidence of stroke and perioperative mortality which was also confirmed by the STABLE trial (42, 43). Therefore, for patients undergoing acute surgery, doctors should pay more attention to the anchoring point of stent implantation during the operation and be highly alert to the occurrence of perioperative stroke.

Reintervention is also an important prognostic indicator. Previous studies did not meta-analyze this index. Except the study that Daniel J et al. clearly pointed out that the 30-day reintervention and long-term reintervention in the acute phase were both more than twice as high as those of the sub-acute group, while the existing other studies showed no significant statistical difference in the re-intervention rate between the two groups. Through the summary of six articles, we reached a similar conclusion to Daniel J et al., namely, there was no significant difference in the re-intervention between the two groups within 30 days, but the long-term re-intervention rate in the group receiving endovascular therapy in the acute phase was 1.42 times as high as that in the subacute phase.

For studies of long-term prognosis, our conclusions seem to be somewhat different. All current studies seem to support the conclusion that the timing of TEVAR for uTBAD does not appear to be independently predictive of or 1-year mortality. By conducted the meta-analysis of published data on long-term survival outcomes, we found no significant difference in long-term mortality between the two group but with significant heterogeneity. This is consistent with the conclusions of published meta-analysis (24). Through data analysis, we suggest that this heterogeneity may be caused by differences in follow-up time. Therefore, we performed a subgroup analysis based on the length of follow-up. After deleting the highly sensitive literature, we found that the mortality rate in the 1-year survival study was much higher in the acute stage group than in the subacute stage group. For longer follow-up studies over 3 or 5 years, the relatively high mortality rate in the subacute phase appears to indicate that the long-term benefit of the acute phase group is superior to that of the subacute phase group. Studies have shown that surgical treatment in the acute phase is a better option because the acute phase of the dissection flap is the softest and provides the best opportunity for remodeling (44). This favorable remodeling also reduces the likelihood of long-term aneurysm degeneration and aortic related mortality (13). Further studies have shown that this relatively good plasticity can be maintained up to 3 months after the initial dissection, while the dissection flap rapidly thickens, straightens, and becomes less flexible within 3 months as aortic wall fibrosis progresses (16, 19, 20). This theory provides favorable support for our results. TEVAR may have the best effect on aortic remodeling in the acute phase after primary dissection, which is reflected in the relatively ideal long-term follow-up results in the acute phase group. However, there are concerns about acute group related with high risky in perioperative complications and increased 30-day and 1-year mortality. In contrast, patients treated in the subacute phase had fewer complications, such as RTAD and aortic rupture. To strike a balance between aortic vulnerability and aortic plasticity, we believe that the subacute phase seems to be a more appropriate window of time to maximize the benefits of early prophylactic TEVAR in patients with TBAD. It remains to be seen whether future technological changes and further risk-benefit assessment can perfectly balance the long-term benefits with the 30-day risk of complications and death.

It is important to note that, as proposed by Torrent et al. (32), the difference in outcomes between the acute and subacute phases may be due in part to the fact that patients in the acute phase represent an inherently higher anatomical or physiological risk population, and this difference cannot be completely eliminated by propensity analysis. With the addition of high-risk characteristics to the new SVS/STS guidelines (7, 26, 28), High-risk aortic dissection is starting to become a separate category. We asked the new questions are patients undergoing acute surgery more likely to have high-risk dissection characteristics? Does the presence of this high-risk dissection have a hidden effect on the conclusions of the existing studies? Potter et al.'s study highlights early intervention has risky for HRF and points out that patients with HRF appear to benefit from at least a short stabilization period prior to TEVAR. At present, there are no other relevant studies on high-risk interlayers. Whether the timing of surgery has a more significant effect on prognosis in patients with high-risk aortic dissection is unknown. The classification of uTBAD or cTBAD according to the characteristics of high-risk dissection marks the gradual maturity of individualized management and refined treatment in clinical practice. Further in-depth study will provide a clearer diagnosis and treatment idea for the management of high-risk patients without complex TBAD. Therefore, in the future, we should pay more attention to the correlation analysis of long-term efficacy and collect more information about the prognostic outcome of patients with high-risk characteristics. More detailed records of risk factors and long-term follow-up data should be used to assess the short-term risk and long-term benefit of different patients to reach the most appropriate individual treatment options.

### 4.1. Limitations

Considering several limitations of the included studies as followed, the results of this meta-analysis should be discussed with caution: (1) So far, the number of relevant studies is small. Due to the limited number of included studies and the existence of studies with small sample sizes, it has difficulties in the analysis of publication bias and heterogeneity in Meta-analysis, which may lead to the conclusion being not robust enough. (2) Because the adjustment factors were not explicitly mentioned in some of the included individual studies, we extracted the original data and conducted a meta-analysis, which may lead to some undetected confounding factors affecting the study conclusions. (3) Since the included studies were all retrospective analyses of single-center experience and lack of randomized controlled studies, our level of evidence was not high enough. (4) There is little data on long-term prognostic outcomes compared with short- and mid-term follow-up data are presented in all studies. It needs to be further updated and improved in the future.

### 4.2. Conclusion

The meta-analysis indicated that 30-day complications, 30-day mortality, reintervention rate and one-year mortality were higher in the acute uTBAD group, but there was no significant difference in long-term follow-up outcomes between the two groups. This meta-analysis confirmed that there was no significant difference in the long-term prognosis between the acute and subacute phases in the timing of surgery. However, considering the high incidence of complications, high re-intervention rate and one-year mortality probably caused by high intima fragility in the acute phase, endovascular repair at subacute phase appears to favorably compare with acute strategy. But future studies with adequate patient numbers and longer-term follow-up are necessary to further verify the study conclusion.

## Data Availability

The original contributions presented in the study are included in the article/**Supplementary Material**, further inquiries can be directed to the corresponding author/s.
