# Supplementary material for: Endovascular repair of acute vs. subacute uncomplicated type B aortic dissection: a systematic review and meta-analysis
Source: Front Cardiovasc Med. 2023 Jul 12;10:1189750. doi: 10.3389/fcvm.2023.1189750 (PMC10369003; doi:10.3389/fcvm.2023.1189750)
Supplement: Supplementary file 2 [file Presentation1.pptx]

## Slide 1
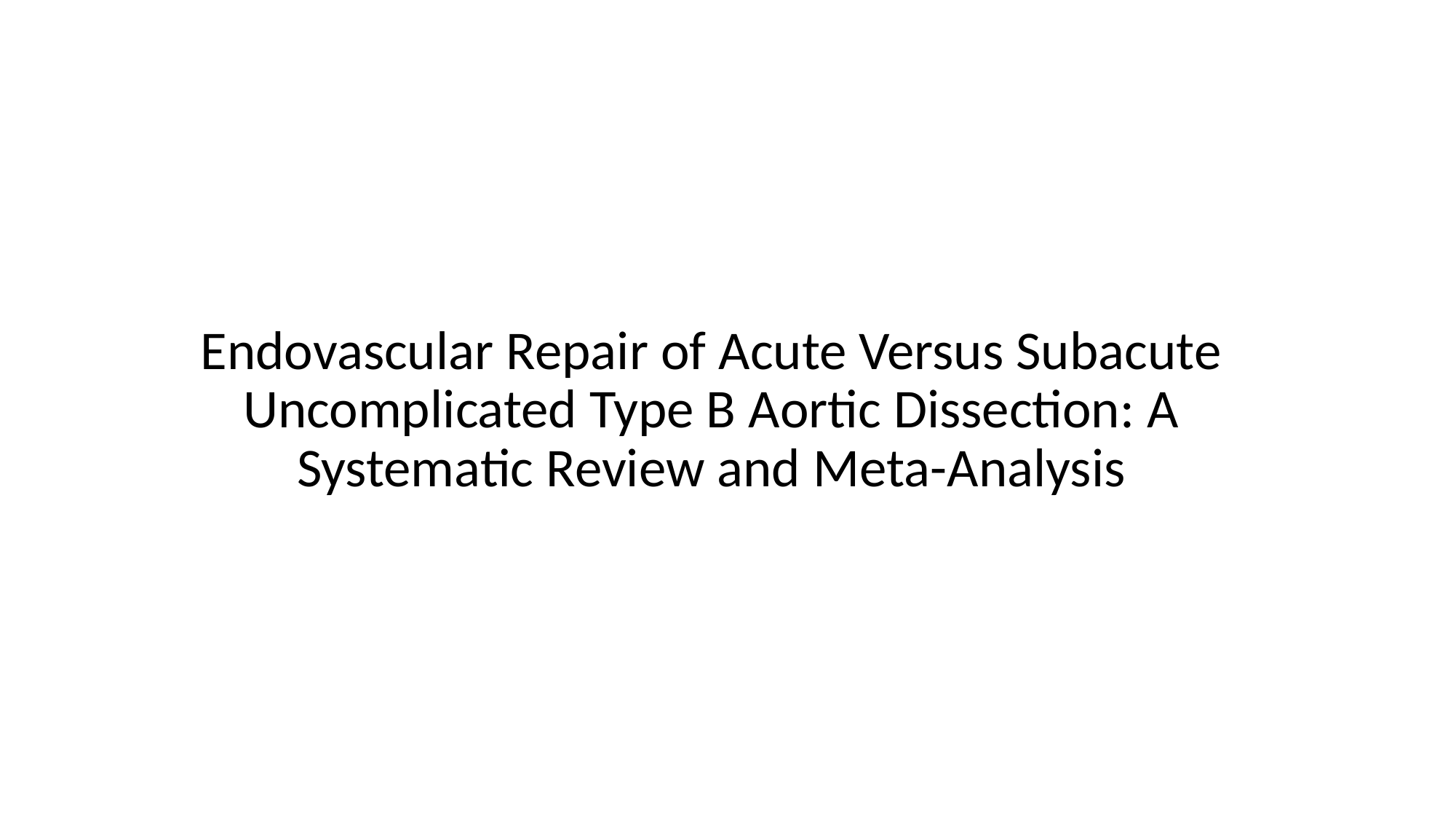

# Endovascular Repair of Acute Versus Subacute Uncomplicated Type B Aortic Dissection: A Systematic Review and Meta-Analysis

## Slide 2
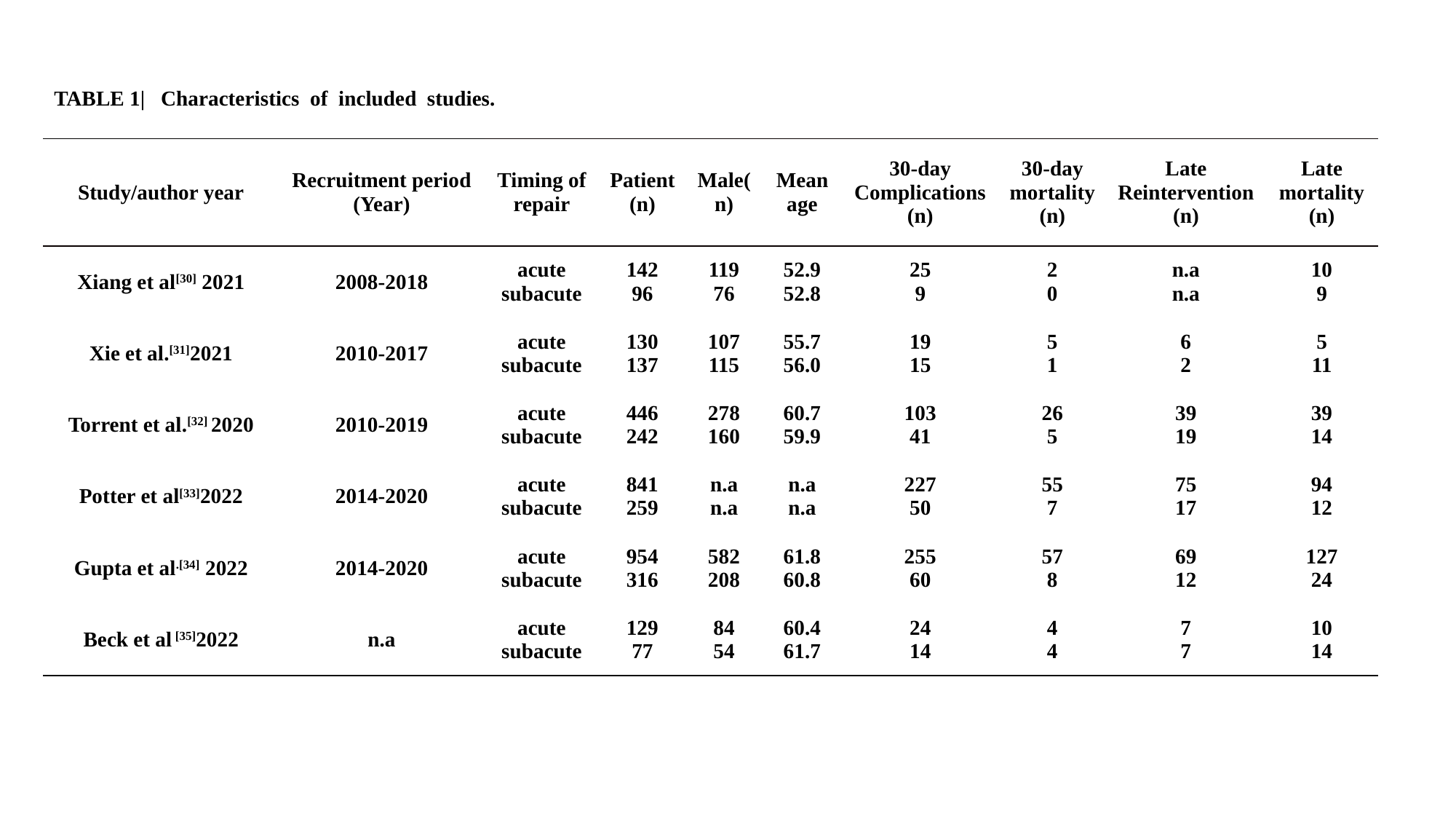

| TABLE 1| Characteristics of included studies. | | | | | | | | | |
| --- | --- | --- | --- | --- | --- | --- | --- | --- | --- |
| Study/author year | Recruitment period (Year) | Timing of repair | Patient(n) | Male(n) | Mean age | 30-day Complications (n) | 30-day mortality (n) | Late Reintervention (n) | Late mortality (n) |
| Xiang et al[30] 2021 | 2008-2018 | acute | 142 | 119 | 52.9 | 25 | 2 | n.a | 10 |
| | | subacute | 96 | 76 | 52.8 | 9 | 0 | n.a | 9 |
| Xie et al.[31]2021 | 2010-2017 | acute | 130 | 107 | 55.7 | 19 | 5 | 6 | 5 |
| | | subacute | 137 | 115 | 56.0 | 15 | 1 | 2 | 11 |
| Torrent et al.[32] 2020 | 2010-2019 | acute | 446 | 278 | 60.7 | 103 | 26 | 39 | 39 |
| | | subacute | 242 | 160 | 59.9 | 41 | 5 | 19 | 14 |
| Potter et al[33]2022 | 2014-2020 | acute | 841 | n.a | n.a | 227 | 55 | 75 | 94 |
| | | subacute | 259 | n.a | n.a | 50 | 7 | 17 | 12 |
| Gupta et al.[34] 2022 | 2014-2020 | acute | 954 | 582 | 61.8 | 255 | 57 | 69 | 127 |
| | | subacute | 316 | 208 | 60.8 | 60 | 8 | 12 | 24 |
| Beck et al [35]2022 | n.a | acute | 129 | 84 | 60.4 | 24 | 4 | 7 | 10 |
| | | subacute | 77 | 54 | 61.7 | 14 | 4 | 7 | 14 |

## Slide 3
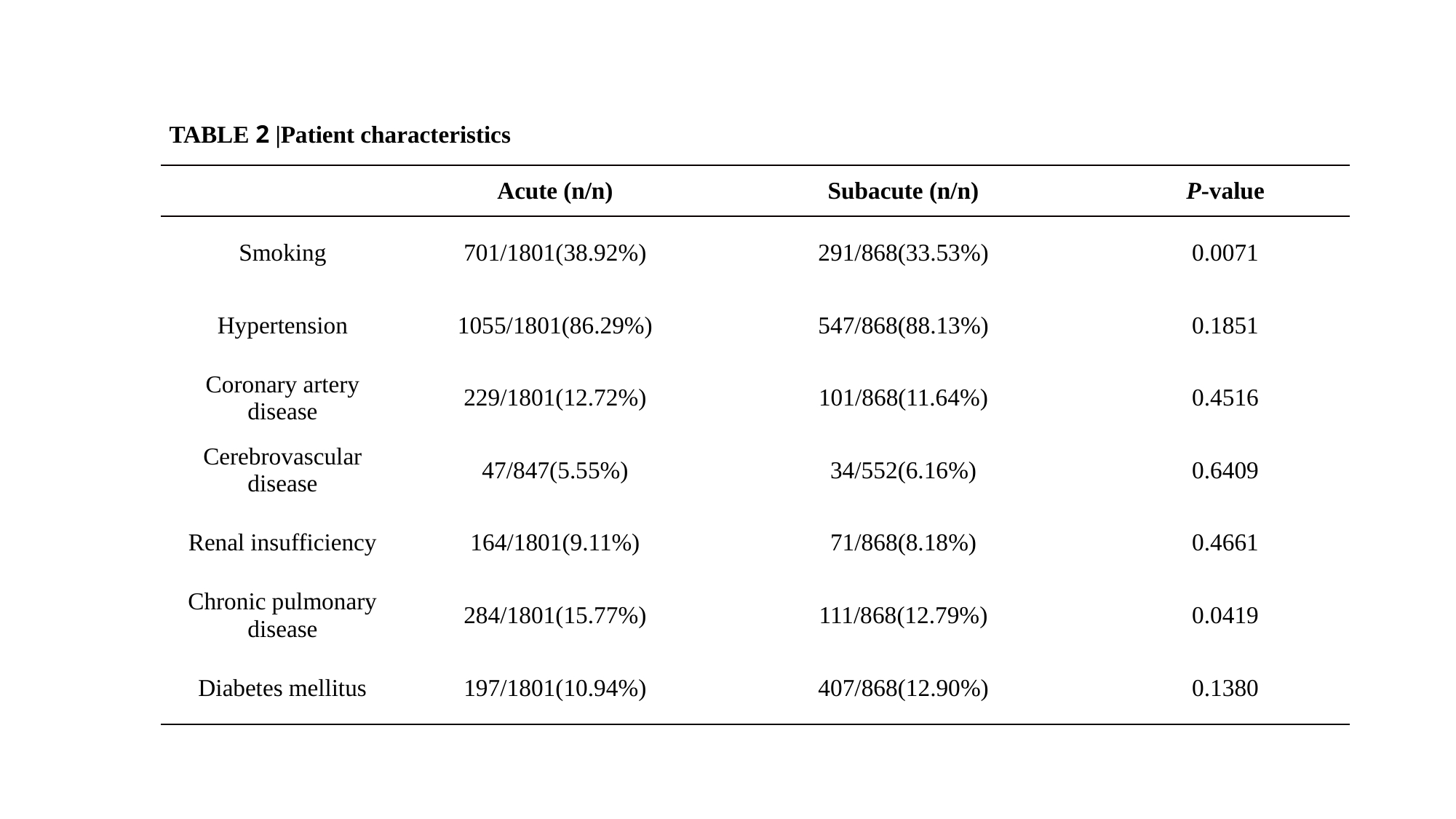

| TABLE 2 |Patient characteristics | | | |
| --- | --- | --- | --- |
| | Acute (n/n) | Subacute (n/n) | P-value |
| Smoking | 701/1801(38.92%) | 291/868(33.53%) | 0.0071 |
| Hypertension | 1055/1801(86.29%) | 547/868(88.13%) | 0.1851 |
| Coronary artery disease | 229/1801(12.72%) | 101/868(11.64%) | 0.4516 |
| Cerebrovascular disease | 47/847(5.55%) | 34/552(6.16%) | 0.6409 |
| Renal insufficiency | 164/1801(9.11%) | 71/868(8.18%) | 0.4661 |
| Chronic pulmonary disease | 284/1801(15.77%) | 111/868(12.79%) | 0.0419 |
| Diabetes mellitus | 197/1801(10.94%) | 407/868(12.90%) | 0.1380 |

## Slide 4
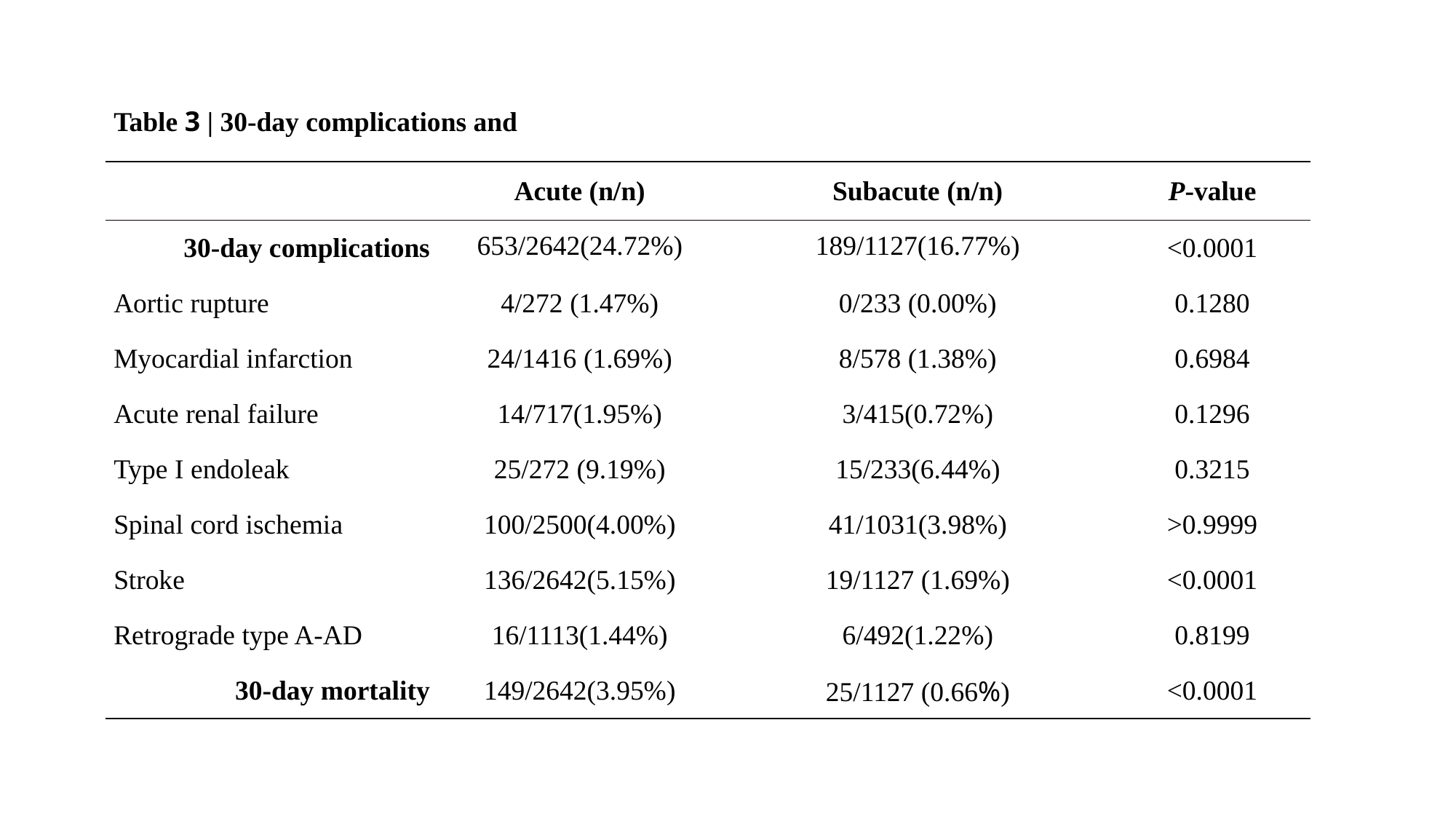

| Table 3 | 30-day complications and | | | |
| --- | --- | --- | --- |
| | Acute (n/n) | Subacute (n/n) | P-value |
| 30-day complications | 653/2642(24.72%) | 189/1127(16.77%) | <0.0001 |
| Aortic rupture | 4/272 (1.47%) | 0/233 (0.00%) | 0.1280 |
| Myocardial infarction | 24/1416 (1.69%) | 8/578 (1.38%) | 0.6984 |
| Acute renal failure | 14/717(1.95%) | 3/415(0.72%) | 0.1296 |
| Type I endoleak | 25/272 (9.19%) | 15/233(6.44%) | 0.3215 |
| Spinal cord ischemia | 100/2500(4.00%) | 41/1031(3.98%) | >0.9999 |
| Stroke | 136/2642(5.15%) | 19/1127 (1.69%) | <0.0001 |
| Retrograde type A-AD | 16/1113(1.44%) | 6/492(1.22%) | 0.8199 |
| 30-day mortality | 149/2642(3.95%) | 25/1127 (0.66%) | <0.0001 |

## Slide 5
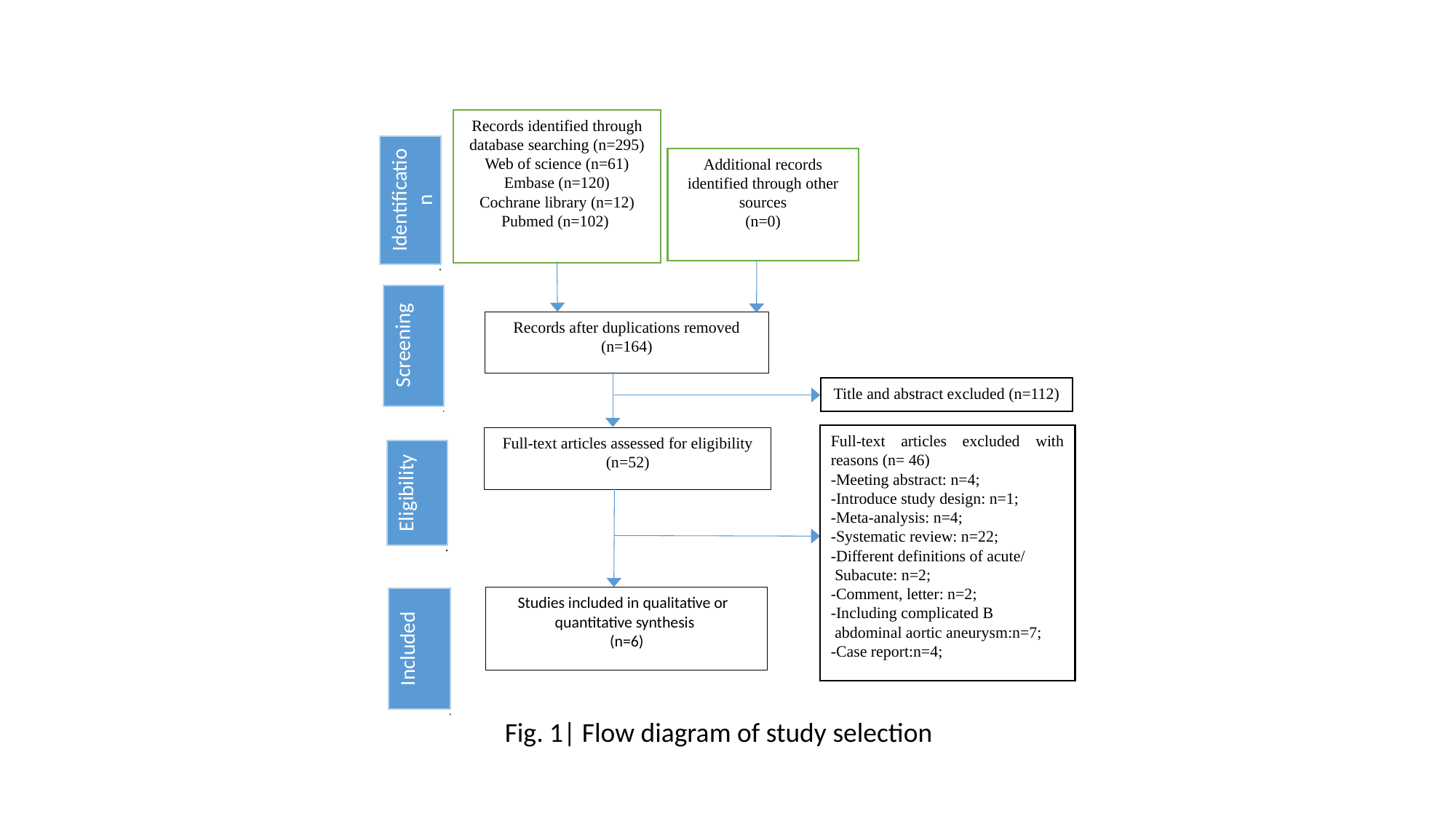

Records identified through database searching (n=295)
Web of science (n=61)
Embase (n=120)
Cochrane library (n=12)
Pubmed (n=102)
Additional records identified through other sources
(n=0)
Records after duplications removed
(n=164)
Title and abstract excluded (n=112)
Full-text articles excluded with reasons (n= 46)
-Meeting abstract: n=4;
-Introduce study design: n=1;
-Meta-analysis: n=4;
-Systematic review: n=22;
-Different definitions of acute/
 Subacute: n=2;
-Comment, letter: n=2;
-Including complicated B
 abdominal aortic aneurysm:n=7;
-Case report:n=4;
Full-text articles assessed for eligibility (n=52)
Studies included in qualitative or quantitative synthesis
(n=6)
Identification
Screening
Eligibility
Included
Fig. 1| Flow diagram of study selection

## Slide 6
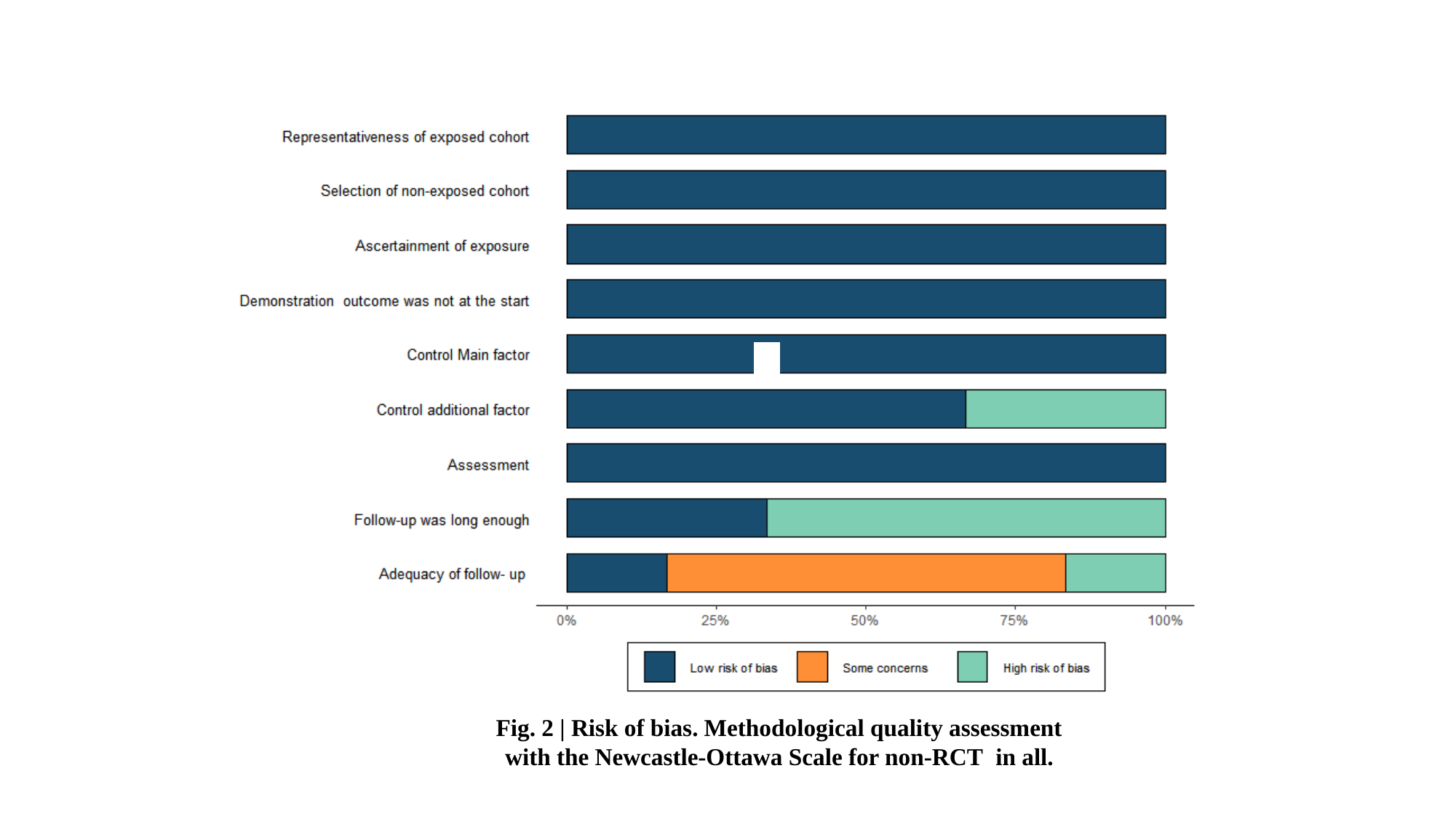

| | |
| --- | --- |
| | |
Fig. 2 | Risk of bias. Methodological quality assessment with the Newcastle-Ottawa Scale for non-RCT in all.

## Slide 7
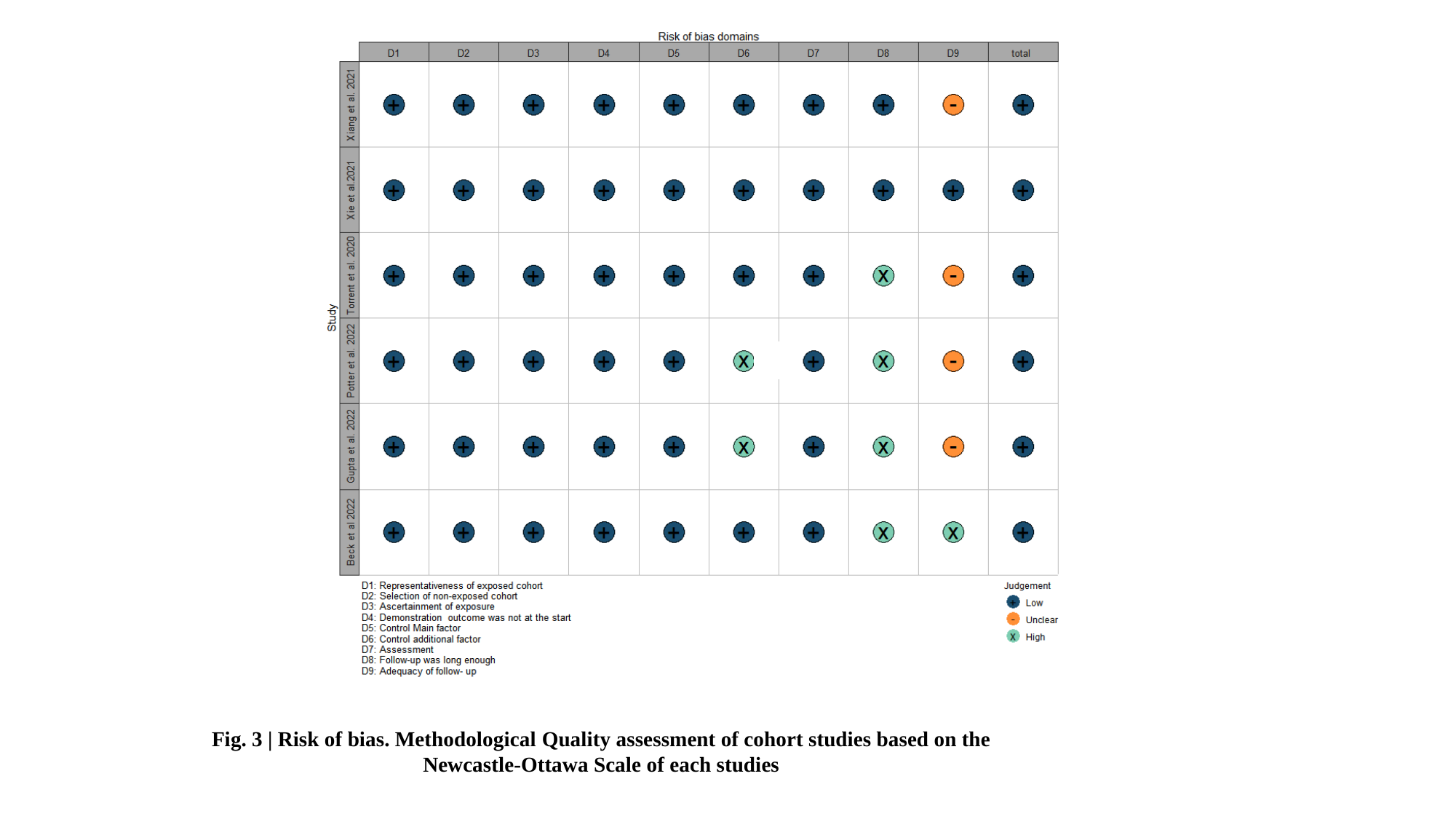

| | |
| --- | --- |
| | |
Fig. 3 | Risk of bias. Methodological Quality assessment of cohort studies based on the Newcastle-Ottawa Scale of each studies

## Slide 8
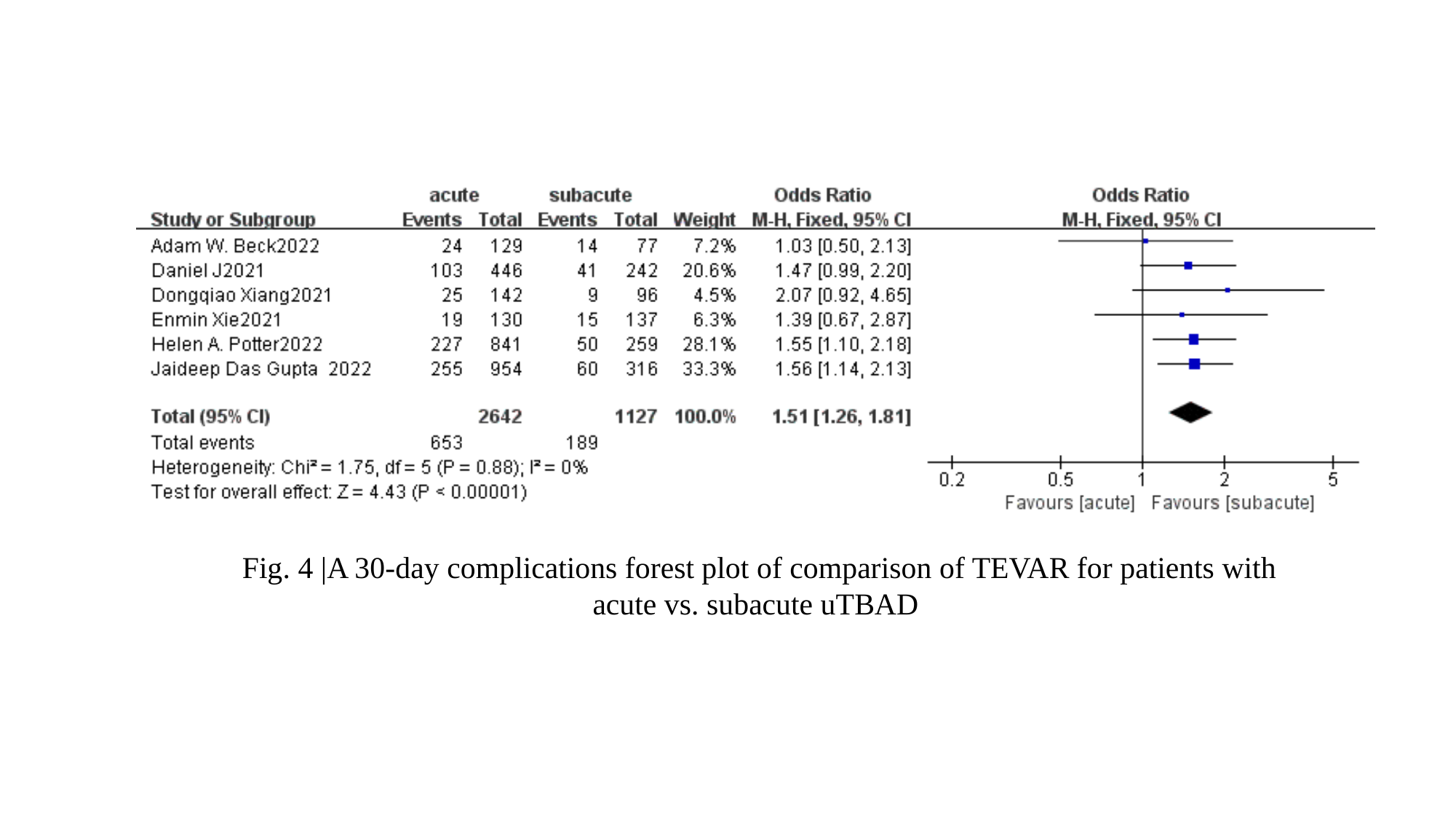

Fig. 4 |A 30-day complications forest plot of comparison of TEVAR for patients with acute vs. subacute uTBAD

## Slide 9
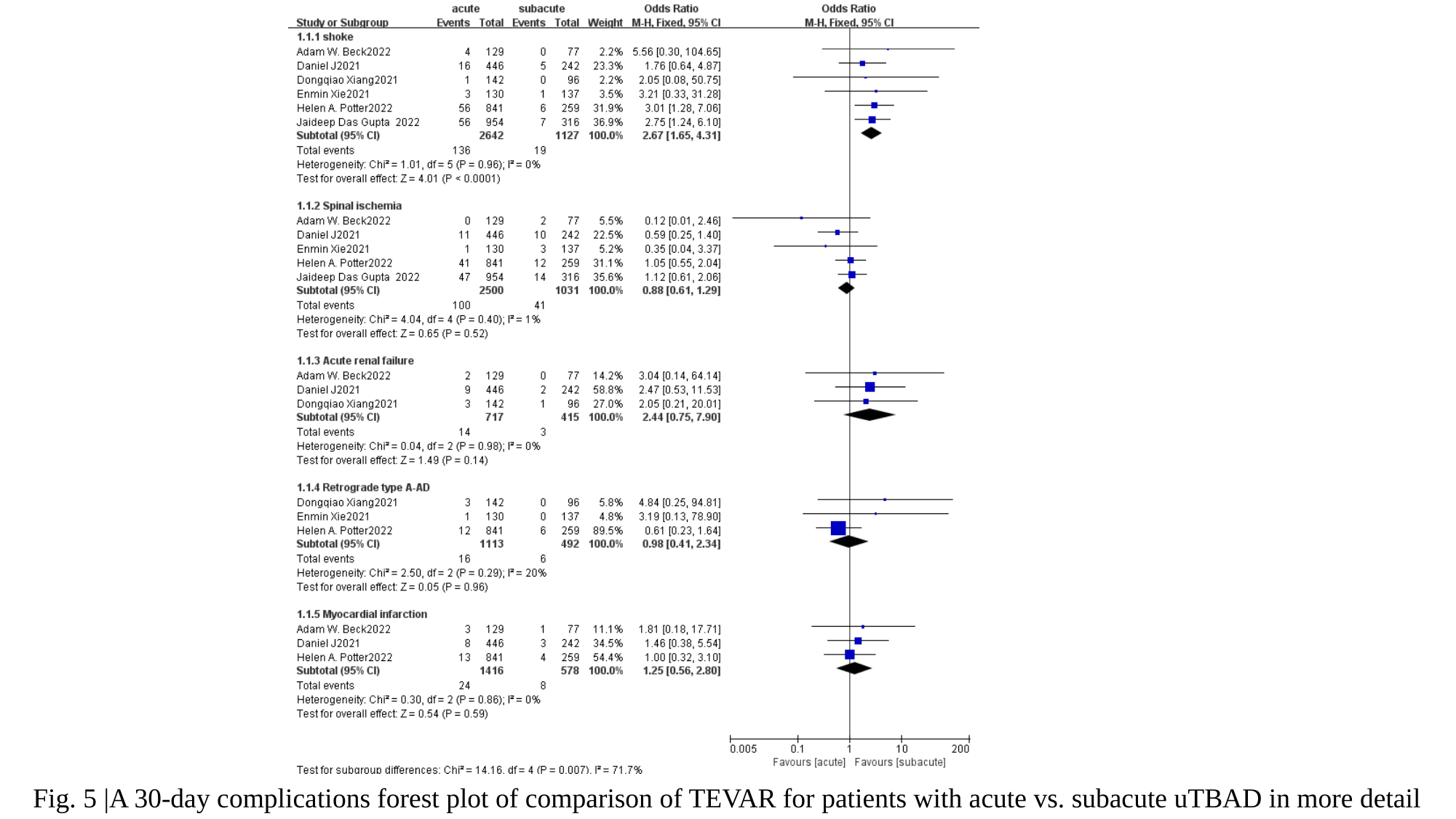

| | |
| --- | --- |
| | |
Fig. 5 |A 30-day complications forest plot of comparison of TEVAR for patients with acute vs. subacute uTBAD in more detail

## Slide 10
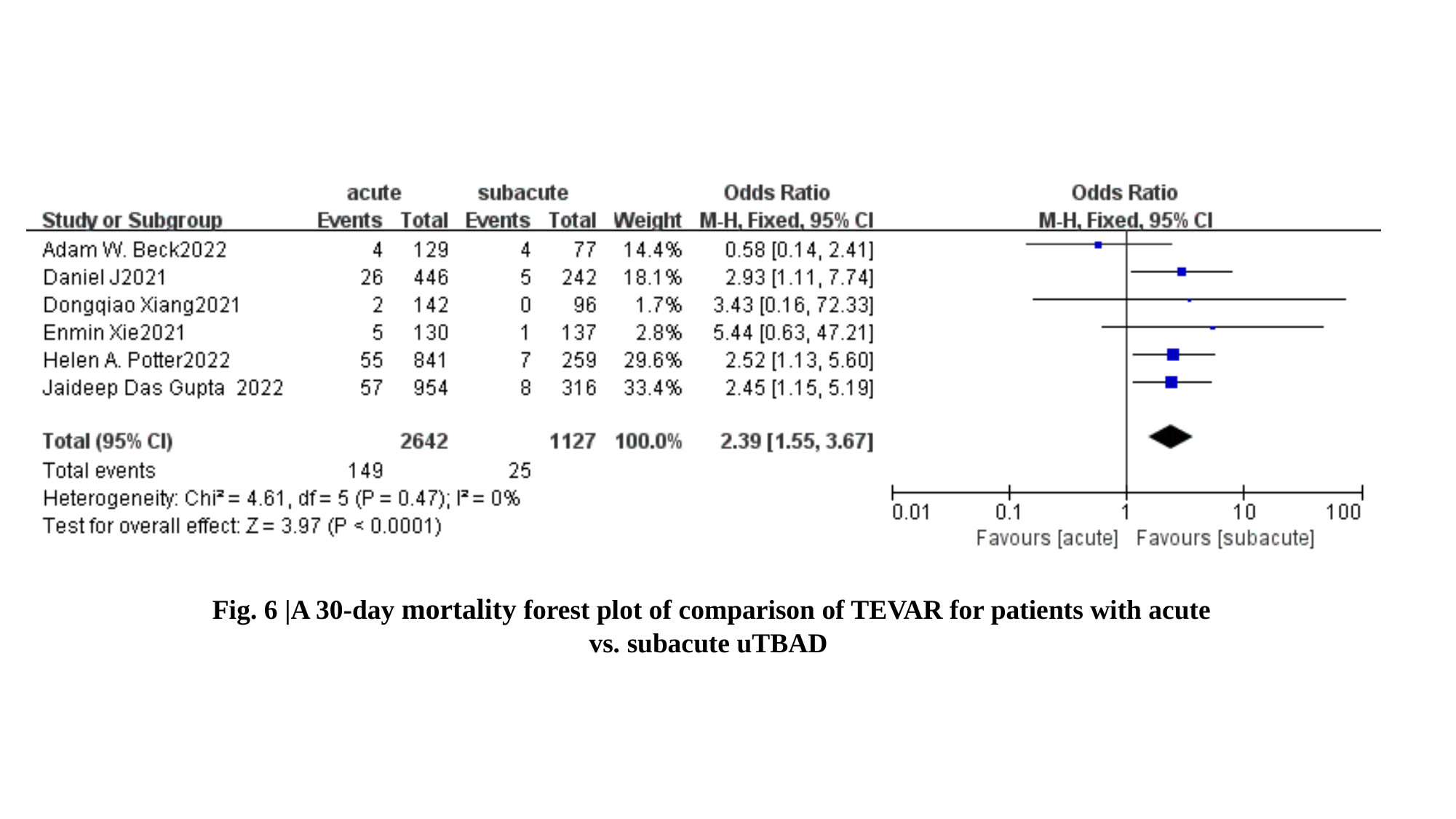

Fig. 6 |A 30-day mortality forest plot of comparison of TEVAR for patients with acute vs. subacute uTBAD

## Slide 11
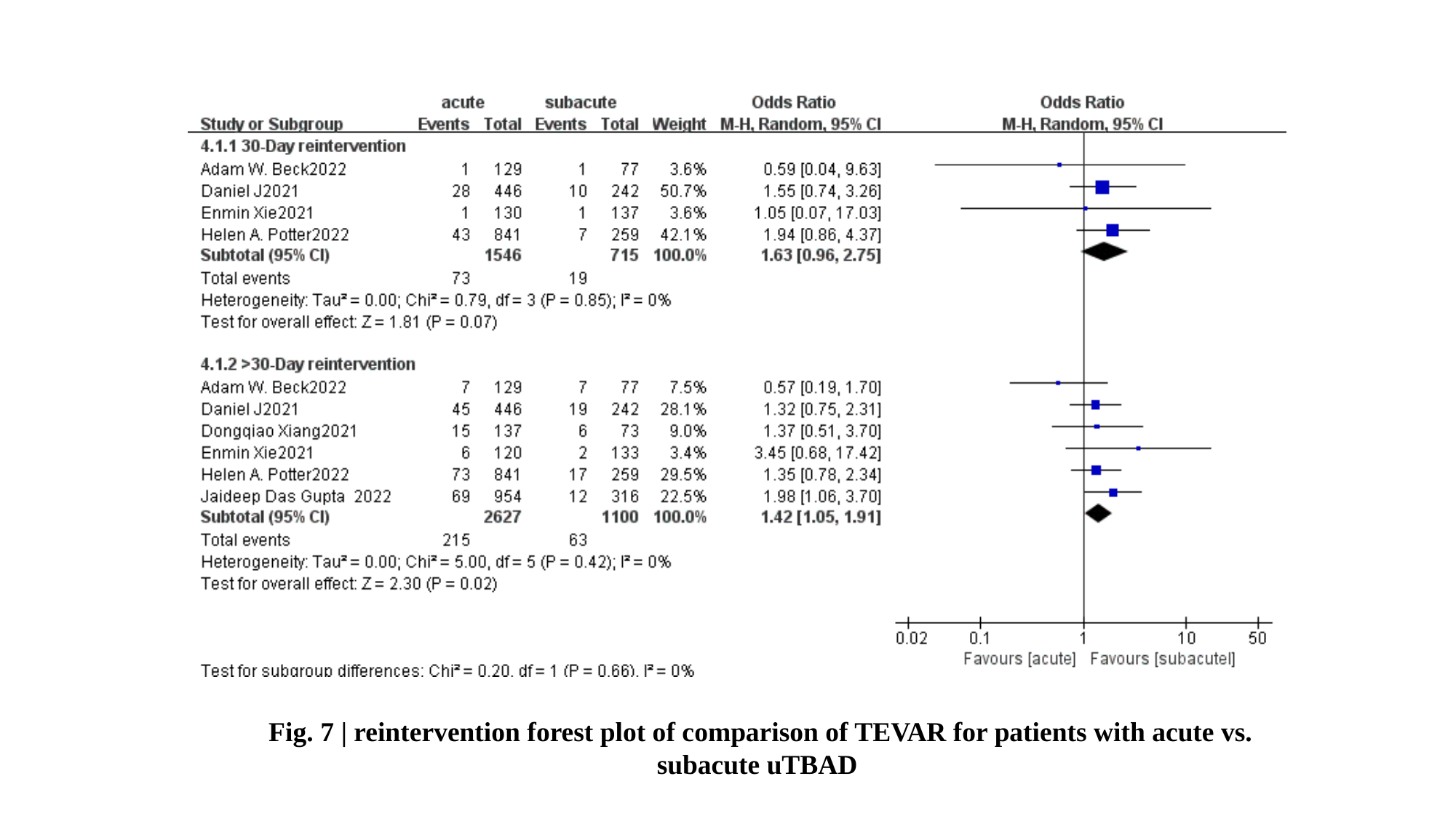

Fig. 7 | reintervention forest plot of comparison of TEVAR for patients with acute vs. subacute uTBAD

## Slide 12
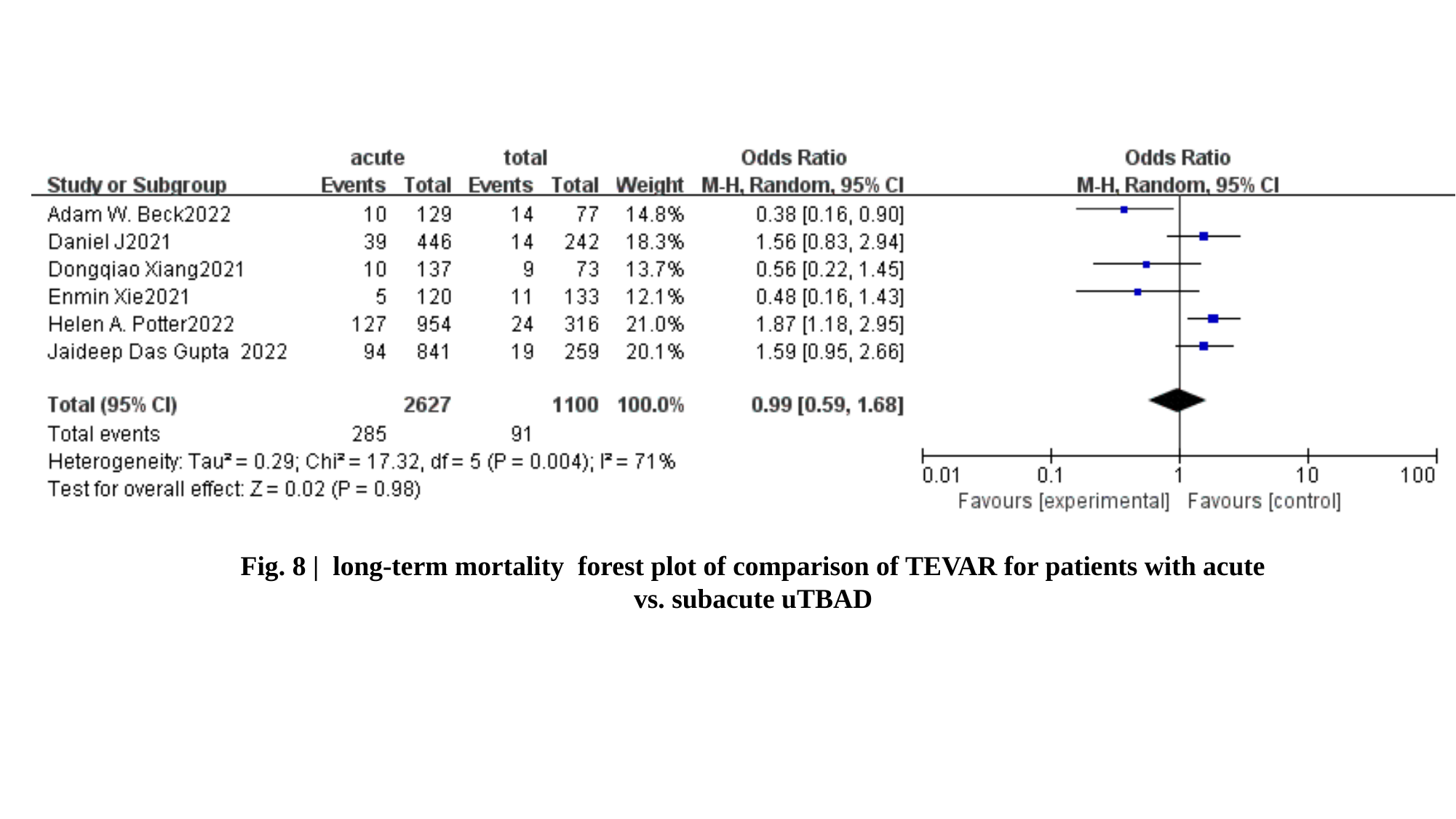

Fig. 8 | long-term mortality forest plot of comparison of TEVAR for patients with acute vs. subacute uTBAD

## Slide 13
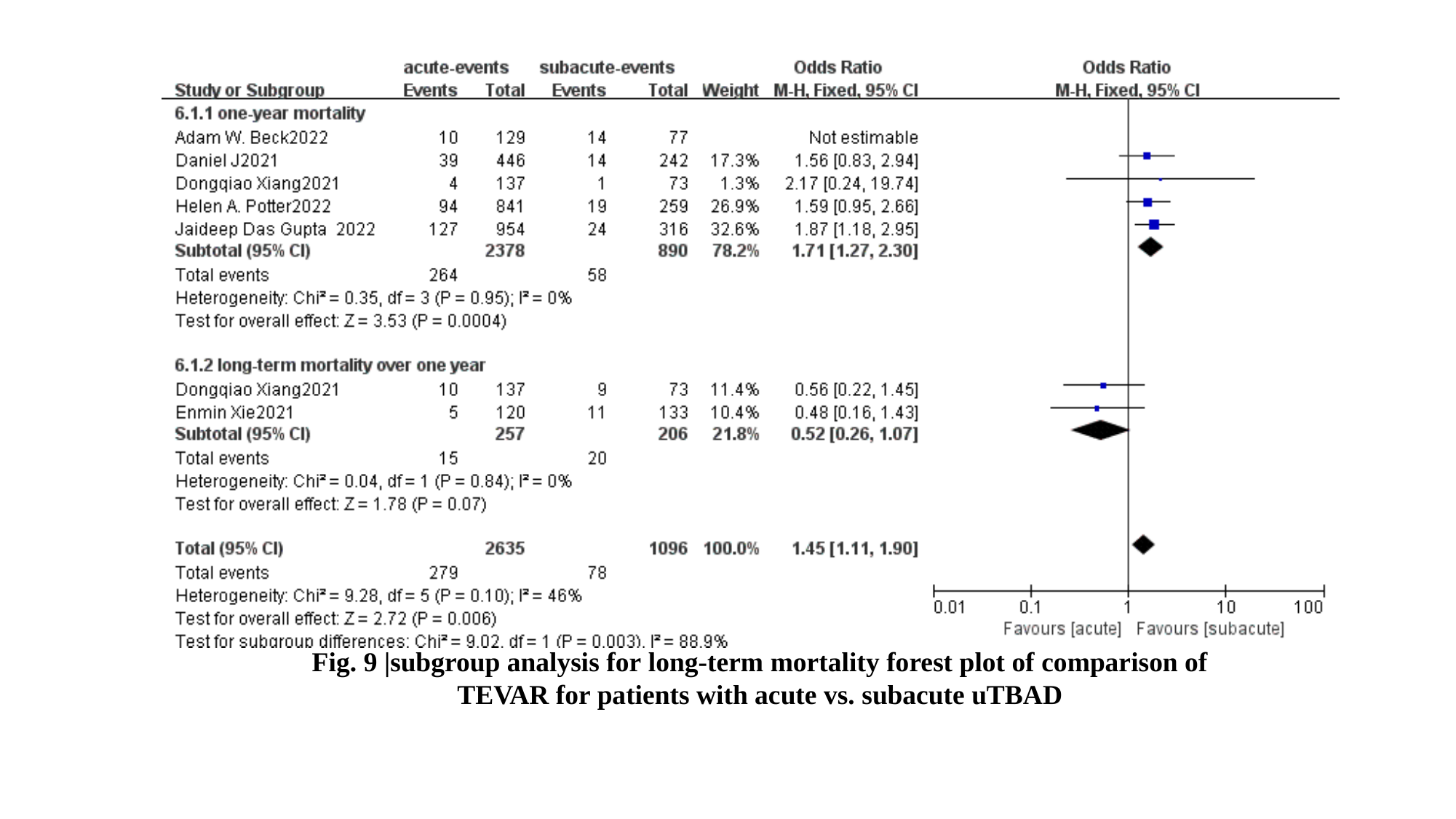

| | |
| --- | --- |
| | |
Fig. 9 |subgroup analysis for long-term mortality forest plot of comparison of TEVAR for patients with acute vs. subacute uTBAD
